# Supplementary material for: ProBASS—a language model with sequence and structural features for predicting the effect of mutations on binding affinity
Source: Bioinformatics. 2025 May 9;41(5):btaf270. doi: 10.1093/bioinformatics/btaf270 (PMC12151015; doi:10.1093/bioinformatics/btaf270)
Supplement: btaf270_Supplementary_Data [file btaf270_supplementary_data.pdf]

Supplementary Information

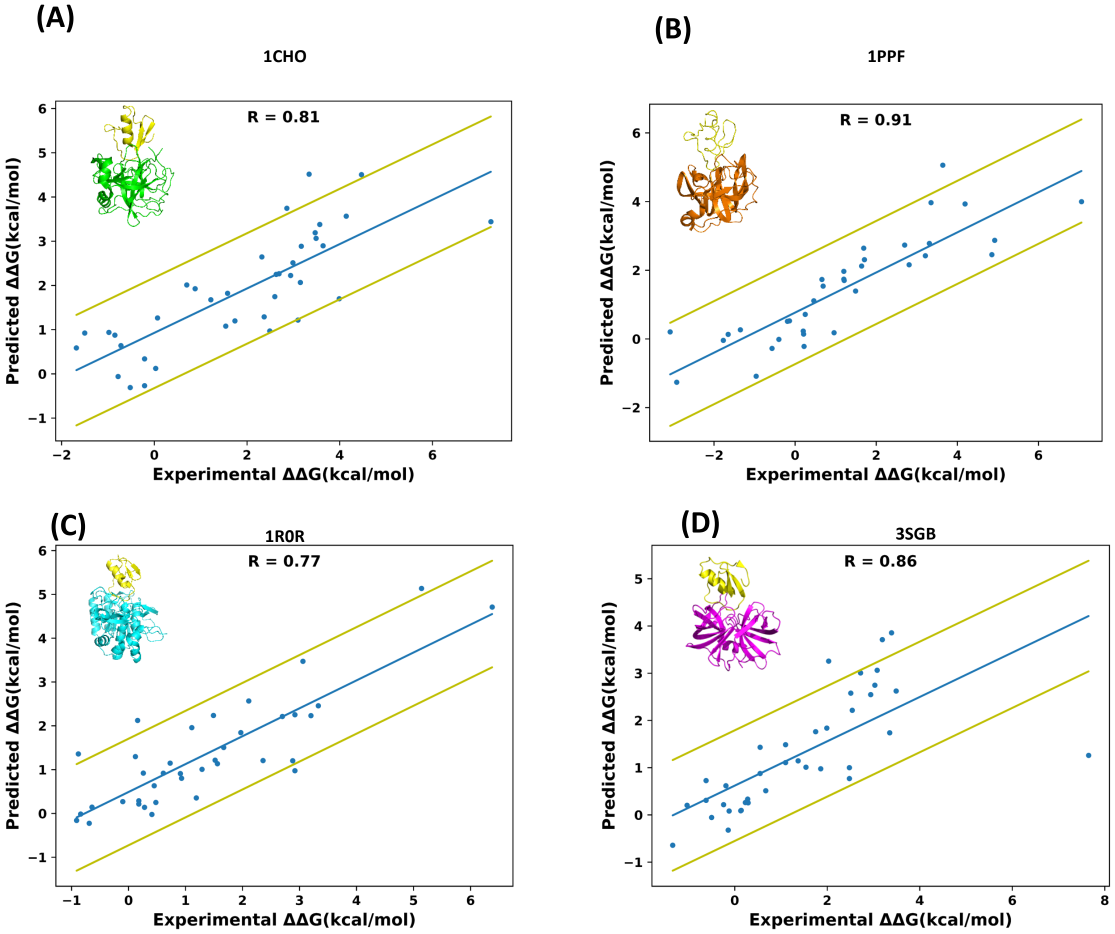

Supplementary Figure 1. Predicting $\Delta\Delta G_{bind}$ in single PPIs. Correlation between experimental and predicted $\Delta\Delta G_{bind}$ values when training and testing is performed on a single PPI: (A) a complex between Turkey Ovomucoid third domain and Alpha-Chymotrypsin (PDB ID 1CHO), (B) a complex between Turkey Ovomucoid third domain and Human Leukocyte elastase (PDB ID 1PPF), (C) a complex between Turkey Ovomucoid third domain and subtilisin carlsberg (PDB ID 1R0R), (D) a complex between Turkey Ovomucoid third domain and subtilisin (PDB ID 3SGB). The blue line represents the best linear fit of the data with the Person correlation R-value given on each graph. The yellow lines represent one standard deviation above and below the fitted line.

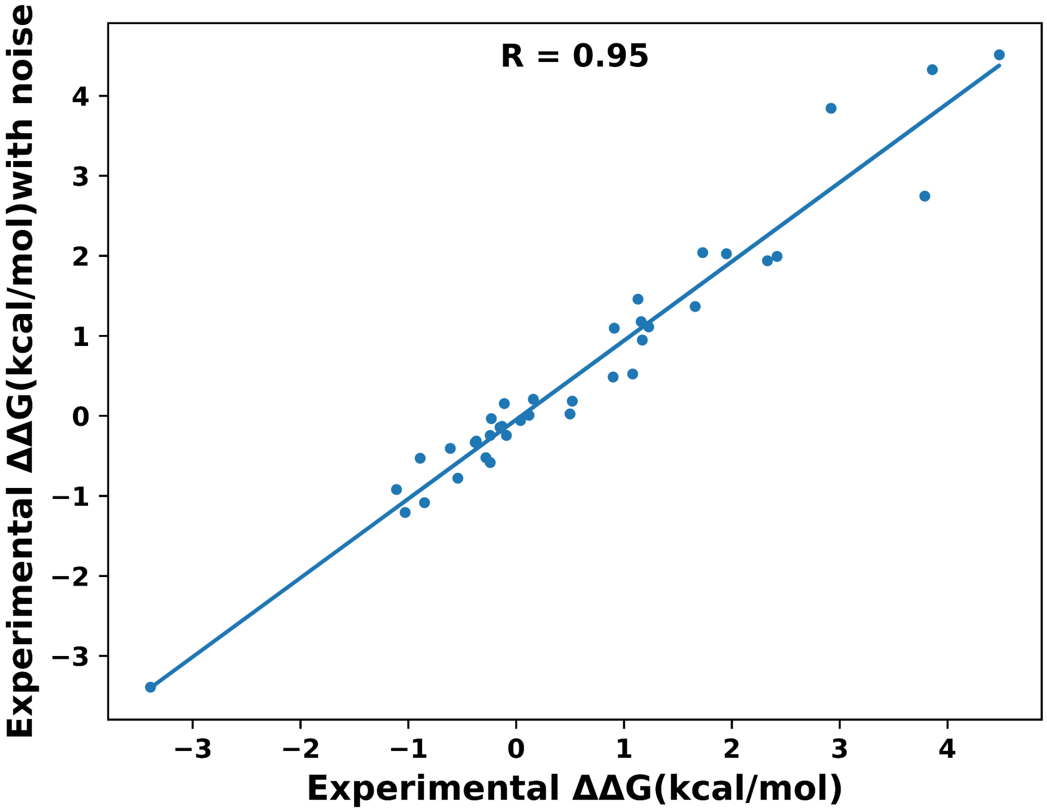

Supplementary Figure 2) Influence of Noise on experimental data. Correlation between experimental $\Delta\Delta G_{bind}$ values and the same values with noise added according to the standard deviation measured for each data point. (data for colicin/DNAse complexes (PBD ID 2WPT and 1EMV).

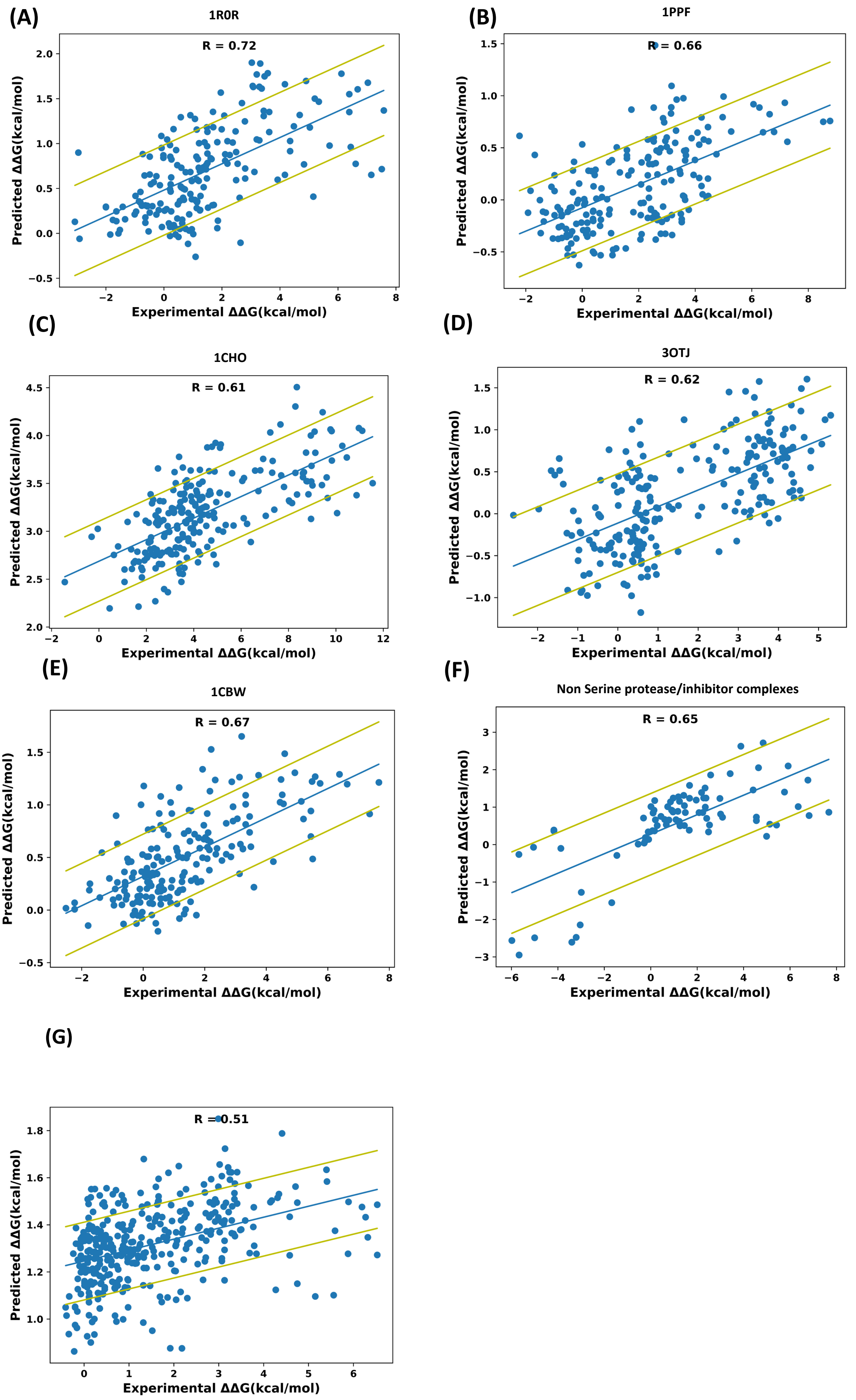
(A)
1R0R
R = 0.72
Predicted ΔΔG(kcal/mol)
Experimental ΔΔG(kcal/mol)
(B)
1PPF
R = 0.66
Predicted ΔΔG(kcal/mol)
Experimental ΔΔG(kcal/mol)
(C)
1CHO
R = 0.61
Predicted ΔΔG(kcal/mol)
Experimental ΔΔG(kcal/mol)
(D)
3OTJ
R = 0.62
Predicted ΔΔG(kcal/mol)
Experimental ΔΔG(kcal/mol)
(E)
1CBW
R = 0.67
Predicted ΔΔG(kcal/mol)
Experimental ΔΔG(kcal/mol)
(F)
Non Serine protease/inhibitor complexes
R = 0.65
Predicted ΔΔG(kcal/mol)
Experimental ΔΔG(kcal/mol)
(G)
R = 0.51
Predicted ΔΔG(kcal/mol)
Experimental ΔΔG(kcal/mol)

Supplementary Figure 3. Predicting $\Delta\Delta G_{bind}$ for single mutations. Correlation between experimental and predicted $\Delta\Delta G_{bind}$ values after the model was trained on the whole dataset excluding the data for the PDB file under evaluation (A) a complex between Turkey Ovomucoid third domain and subtilisin carlsberg (PDB ID 1R0R), (B) a complex between Turkey Ovomucoid third domain and Human Leukocyte elastase (PDB ID 1PPF), (C) a complex between Turkey Ovomucoid third domain and Alpha-Chymotrypsin (PDB 1CHO), (D) a complex between BPTI and bovine Trypsin (PDB ID 3OTJ), (E) a complex between BPTI and Chymotrypsin (PDB ID 1CBW). (F) Non serine protease/inhibitor complexes (PDB IDs : 1CSE, 1CT2, 1EMV, 1S1Q, 1SBB, 1SGD, 1CT2). The blue line represents the best linear fit of the data. (G) A complex between Angiotensin-converting enzyme 2 (ACE2) and Spike protein S1 (PDB ID 6M0J). The yellow lines represent one standard deviation above and below the fitted line.

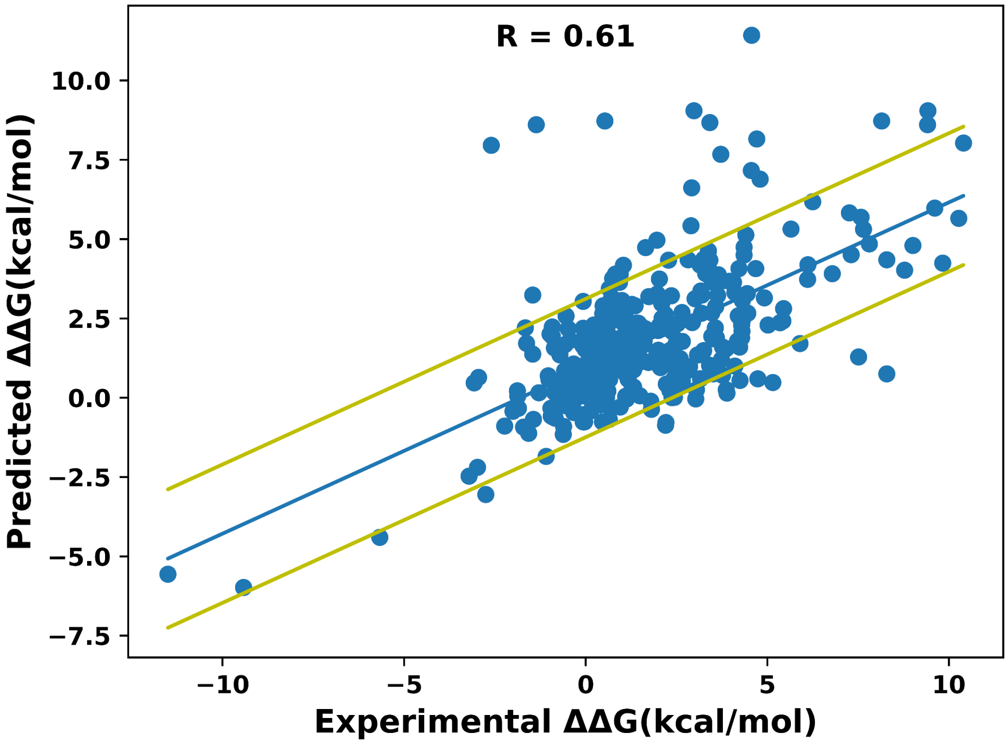

Supplementary Figure 4. Correlation between experimental and predicted $\Delta\Delta G_{bind}$ values when training and testing was performed on the whole dataset of single mutations without Structural embeddings (Embeddings from model ESM-IF1). Mutations were randomly allocated into training and testing sets (80% and 20% of data points, respectively), allowing mutations from the same PDB to potentially appear in both sets. The blue line represents the best linear fit of the data. The yellow lines represent one standard deviation above and below the fitted line.

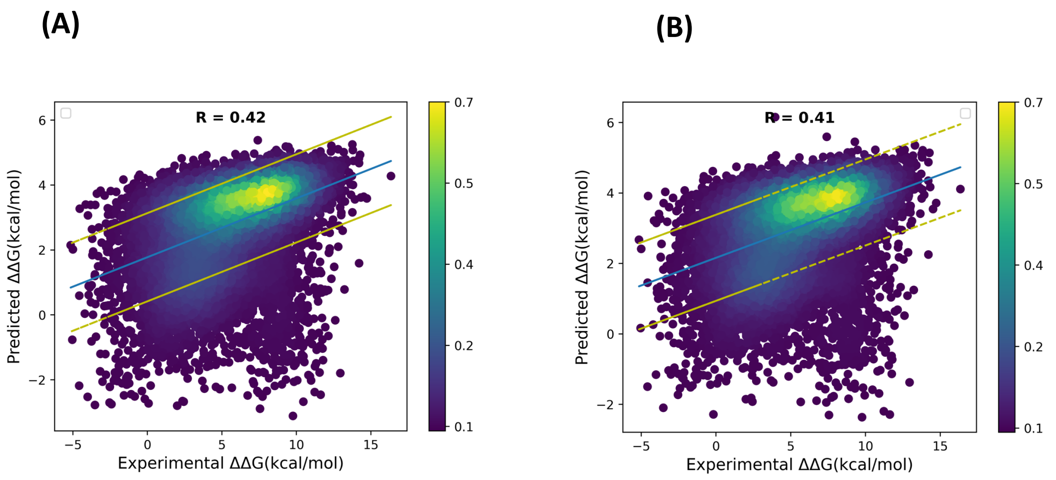

Supplementary Figure 5. Predicting $\Delta\Delta G_{bind}$ for double mutations (A) Correlation between experimental and predicted $\Delta\Delta G_{bind}$ when model was trained on double mutations belonging to the BPTI /Chymotrypsin complex (PDB ID 1CBW) and tested on double mutations belonging to the BPTI/bovine Trypsin complex (PDB ID 3OTJ). (B) Correlation between experimental and predicted $\Delta\Delta G_{bind}$ when model was trained on the whole dataset of double mutants and tested on double mutations belonging to the BPTI/ bovine Trypsin complex (PDB ID 3OTJ). The blue line represents the best linear fit of the data. The yellow lines represent one standard deviation above and below the fitted line. The points are colored according to their local density, with the color bar indicating the density scale. Higher density areas (yellow color) represent regions where data points are more concentrated.

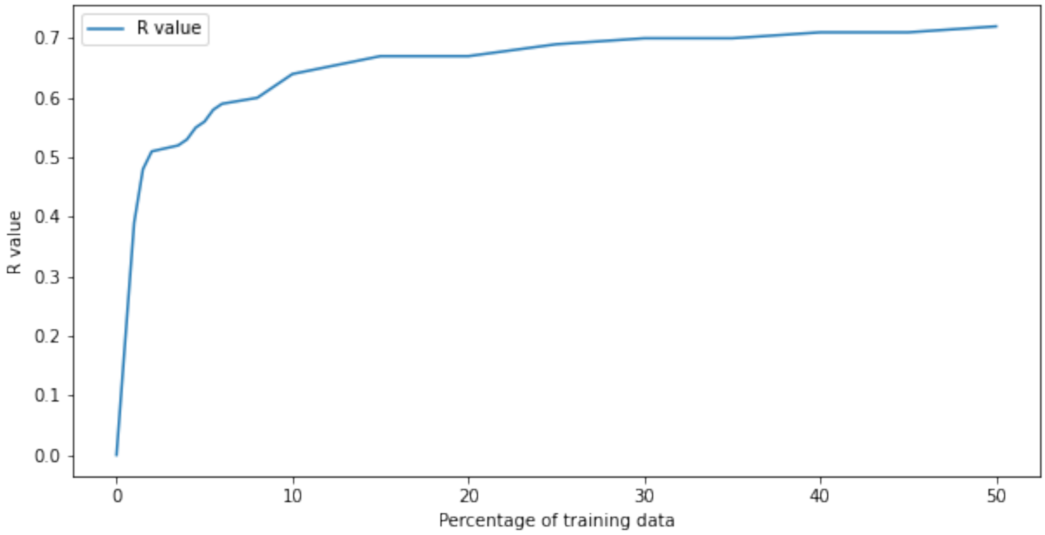

Supplementary Figure 6. Training data vs Correlation. Graph illustrates the impact of increasing the percentage of training data on the R value between experimental and predicted $\Delta\Delta G_{bind}$.

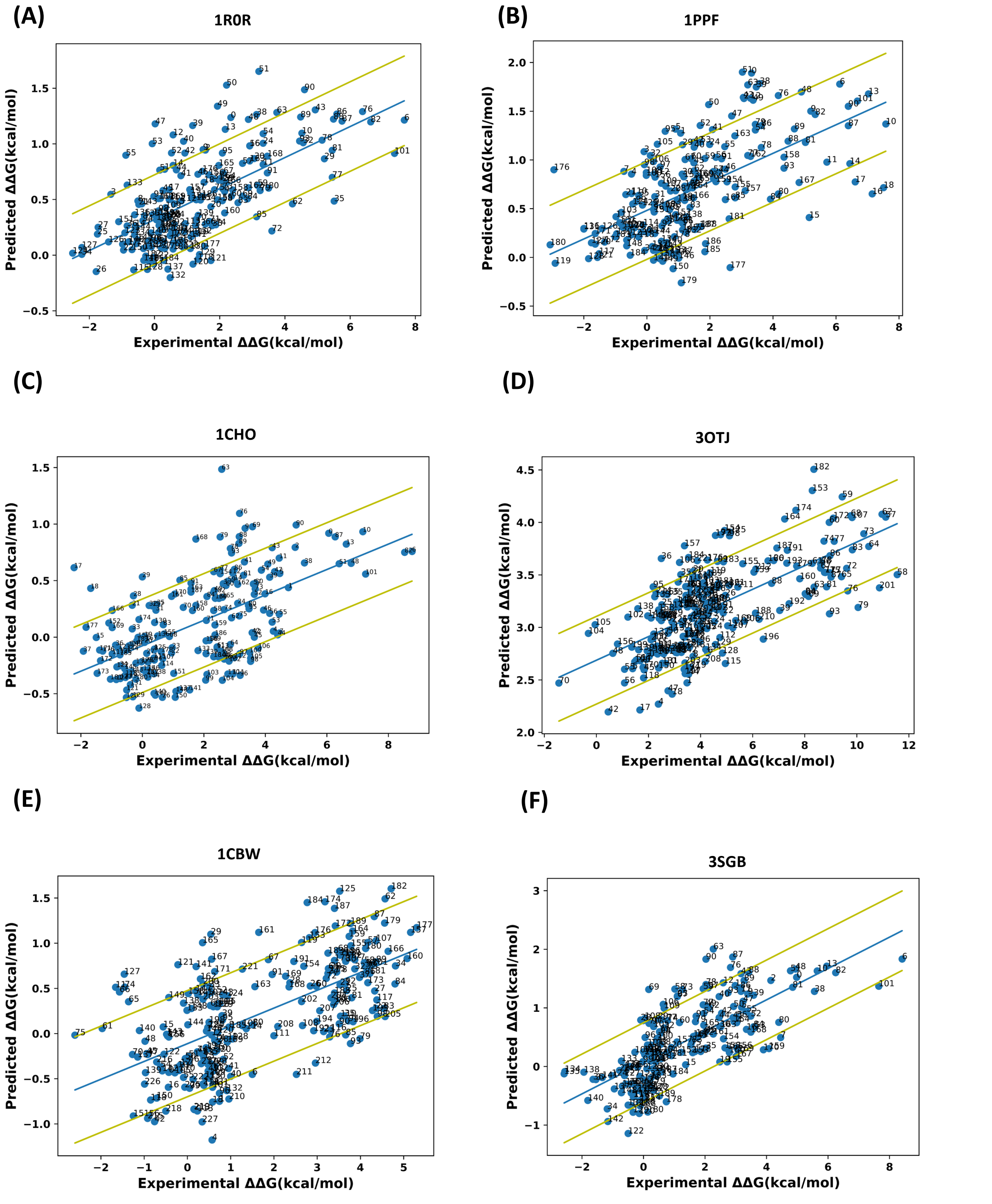

Supplementary Figure 7. Analysis of the outliers for the six PDBs (A) PDB ID 1R0R, (B) PDB ID 1PPF, (C) PDB ID 1CHO, (D) PDB ID 3OTJ, (E) PDB ID 1CBW, (F) PDB ID 3SGB. ProBASS was trained on the whole dataset excluding the test PDB file and predictions were made. The blue line represents the best liner fit to the data and the yellow lines correspond to one standard deviations from the fitted line. The mutations lying above and below the one-standard-deviation line were numbered and analyzed in the context of the structure. See

Supplementary data for mutation description, where mutations predicted to be overly disruptive to the complex are colored in red and mutations predicted overly stabilizing for the PPI are colored in cyan. Outliers. Xlxm file is available in the ProBASS repository (https://github.com/sagagugit/ProBASS).

No of mutations in protease/inhibitor complexes	Total no of mutations	Percentage of mutations in protease/inhibitor complexes	Correlation between prediction and experiment for the test dataset
1020	2320	43.9	0.81
830	2130	38.9	0.79
602	1902	31.6	0.73
374	1674	22.3	0.69
184	1484	12.3	0.7
150	1450	10.3	0.68

Supplementary Table 1. Dependence of correlation coefficient on the number of mutations belonging to the protease/inhibitor PDBs. For training we used the whole set of single mutations while gradually reducing the number of mutations bellowing to Protease/inhibitor complexes. For testing the performance, we used the mutations belonging to the PDB file 3SGB.
